# Notions About Men and Masculinities Among Health Care Professionals Working With Men’s Sexual Health: A Focus Group Study

**DOI:** 10.1177/15579883221101274

**Published:** 2022-06-21

**Authors:** Tommy Persson, Jesper Löve, Ellinor Tengelin, Gunnel Hensing

**Affiliations:** 1 School of Public Health and Community Medicine, Institute of Medicine, Sahlgrenska Academy, University of Gothenburg, Gothenburg, Sweden; 2 Region Västra Götaland, Knowledge Center for Sexual Health, Gothenburg, Sweden; 3 Department for Health Sciences, University West, Trollhättan, Sweden

**Keywords:** masculinity, qualitative research, focus groups, sexual health, men’s health

## Abstract

Health care professionals’ (HCPs) notions about gender may influence the provision and quality of care. If care-seeking men are met by HCPs holding idealized and stereotypical notions of masculinity, this could reinforce barriers to adequate care. This study explored notions about men and masculinities among HCPs working with men’s sexual health in Sweden. Focus group interviews with 35 HCPs from primary health and sexual health clinics were analyzed using qualitative content analysis. The analysis resulted in three descriptive themes: (a) *Contradictory masculinity—elusive but clear*. Notions of masculinity as a phenomenon or concept were elusive, but masculine and un-masculine traits, behaviors, and qualities were clear. (b) *Sexual health care is a social place where men and masculinities can be challenging*. Male patients were associated with unwanted sexual tensions. Masculinity could challenge professionality. Seeking sexual health care was perceived as doing un-masculinity. (c) *Regarding masculinity as irrelevant—a difficult ambition to achieve*. Participants strived for gender-neutrality by regarding patients as humans, individuals, or patients rather than as men and masculine. The analysis also identified a theme of meaning: *Notions of masculinity are situated relationally*. HCPs situate masculinity in real and hypothetical relationships. Romantic and sexual preferences were used to define preferred masculinity. This study identified themes that showed how HCPs balanced professional and private notions of men and masculinity in their patient encounters. Increased gender awareness and training are needed to professionalize the management of gendered notions in encounters with men who seek care for sexual health problems.

## Introduction

Gender is a sociocultural construct that cannot be equated with biological sex or the dichotomies of male and female, and that affects health and well-being on personal, institutional, and structural levels (Heise et al., 2019). In health care organizations, taken-for-granted notions about gender may influence the provision of care (Hay et al., 2019), and health care professionals (HCPs) may construct and reproduce gender norms in their work (Courtenay, 2000). Previous studies have repeatedly suggested that such norms negatively affect the quality of health care provided to women (Hamberg et al., 2004; Nyberg et al., 2008; Stålnacke et al., 2015). Similarly, gender norms also seem to affect men’s health (Gupta et al., 2019), health care practice (Seymour-Smith et al., 2002), and health care communication (Foss & Sundby, 2003; Himmelstein & Sanchez, 2015). Awareness of gender notions and norms is therefore important for health care organizations providing adequate care and for counteracting gender-based inequities in health and care (Celik et al., 2011; Lindsay et al., 2019).

Although there are various examples of men and masculinity being regarded as the norm in health care (Holge-Hazelton & Malterud, 2009; Shaw et al., 2017; Travis et al., 2012), some health care sectors appear to be geared toward women and children (Banks, 2001). One such sector is sexual health care, which has been suggested to be inadequate in meeting men’s health care needs (Collumbien & Hawkes, 2000; Kalmuss & Tatum, 2007; Porche, 2012). Previous studies have revealed that normative masculinity can be a barrier to men’s care-seeking (Smith et al., 2006; Vogel et al., 2011). These studies have mainly focused on the link between men’s endorsement of masculinity norms and their reluctance to seek care as opposed to other barriers to men’s access to sexual health care (Farrimond, 2012; Galdas et al., 2005; O’Brien et al., 2005; Vogel et al., 2011).

Masculinity norms provide a framework for how men are expected to be, behave, and think within a social setting (Liefbroer & Billari, 2010; Rimal & Lapinski, 2015; Rivera, 2020). These norms contribute to hegemonic masculinity that refers to culturally dominant and stereotypical masculinity (Connell & Messerschmidt, 2005; Lee & Owens, 2002; Smith, 2012). Hegemonic masculinity can be said to be both personal and collective projects (Connell & Lindén, 2008; Hearn & Morrell, 2012). On a collective level, it is a normative structure that differentiates power between groups of men and gives men power over women. On a personal level, it is normative patriarchal notions of men and socially acceptable practices of manliness that a person enacts to pass as a biological man within a culture or society (Connell & Messerschmidt, 2005; Courtenay, 2000; Lee & Owens, 2002). Hegemonic masculinity places expectations on men to have certain traits, which may vary over time and between contexts. In research, hegemonic masculinity in contemporary Western culture is generally associated with being heterosexual, white, middle class, independent, robust, physically competent, and emotionally restrictive (Evans et al., 2011; Hearn & Morrell, 2012; Rivera, 2020).

Notions of hegemonic masculinity can create barriers to care for both the patient and HCP. For example, a perceived homophobic context can make it difficult for gay, bisexual, and transgender men to discuss their sexual health, and heterosexual men can incorrectly be considered low risk regarding STIs (sexually transmitted infections) and HIV, based on their identity as heterosexual rather than their sexual practice (R. Knight et al., 2013). In this example, notions about patients’ identity create barriers both toward men who adhere to hegemonic masculinity and men who challenge it. Notions such as these can inform and reinforce prejudice (Crandall et al., 2002; Pereira et al., 2009) and individuals who have experienced prejudice are more likely to refrain from seeking medical treatment (Wamala et al., 2007). There is a lack of studies exploring notions among HCP in sexual health care that could create barriers to adequate care for men seeking sexual health care. As masculinity norms are generally reproduced and created in interactions between persons and institutions, it is vital to understand what notions this interaction entails. Such understanding could be important for eliminating barriers and for enhancing the quality of care received by men seeking sexual health care.

This study aims to explore notions about men and masculinities among HCPs working with men’s sexual health in Sweden. Specifically, we explored how HCPs think about and describe men and different forms of masculinity from a professional standpoint, and we also explored what HCPs perceive to be masculine and un-masculine traits and behaviors. Implications for training and organization of care are discussed.

## Method

### Study Design

Departing from the explorative aim, the study was designed as a qualitative focus group study. This method is well suited to gathering different perspectives on a subject (Krueger, 2009), filling in gaps in understanding and drawing out complex, nuanced, and even contradictory perceptions about a subject (Kamberelis & Dimitriadis, 2014). The study was approved by the Regional Ethical Review Board in Gothenburg, Sweden (Registration No. 543-14).

### Recruitment

To obtain a broad variation of clinical experience of men’s sexual health, we invited HCPs from both primary health care centers (PHCCs) and clinics specializing in sexual and reproductive health. We aimed for different types of clinics, occupational categories, and different clinic catchment areas. The inclusion criterion for HCPs was that they should be working clinically with men’s sexual health. We assumed that HCPs would be more comfortable sharing and discussing their notions with co-workers, and invited all staff at specific clinics to participate. The clinics were recruited through the Sexual and Reproductive Health and Rights Network in Region Västra Götaland (SRHR Network) in southwestern Sweden. The network is interorganizational and aims to develop and co-ordinate strategies to promote sexual and reproductive health and rights. Through this network, we contacted representatives from sexual and reproductive health clinics such as STI clinics, antenatal clinics, clinics of gynecology and venereology, abortion clinics and youth clinics, and PHCCs. A letter with information about the study was sent to key stakeholders in four geographically organized subnetworks that make up the SRHR Network, asking them to pass it on to heads of clinics whose HCPs fulfilled the inclusion criteria. The letter was also published in the network’s newsletter. All letters encouraged those interested in participating to contact the first author (T.P.) via email. Those who did received more information about the study and written consent forms to distribute to all interested HCPs. They were also informed about their right to withdraw from the study at any time.

Nine clinics expressed interest in participating, of which three ultimately chose not to participate due to time constraints. None of the interested clinics were PHCCs. As the availability of clinics specializing in adult men’s sexual health is limited and PHCCs are the main provider of these services, we deemed it important to include at least one such clinic. Thirty PHCCs outside the SRHR Network were contacted directly. Unfortunately, none of them opted to participate. A new letter was sent to the SRHR Network asking them to send it to PHCCs in their geographical area. This resulted in one PHCC choosing to participate. In total, 35 individuals participated from seven different clinics.

### Focus Group Procedure

Seven focus groups were carried out with four to six participants per group. All participants provided written informed consent before participating in the focus group session. The sessions were approximately 90 min long and were conducted at the clinics in rooms that would ensure privacy. The participants were informed that they could contact the first author if they had questions about the study or their participation. The focus groups were moderated by the first author and co-moderated by four different researchers within public health. Two had extensive experience with focus group interviews, two had clinical backgrounds, and one had extensive experience working with sexual health. The co-moderators had no involvement in the study beyond data collection. Their assignment was to identify gaps and contradictions, and to help explore lines of reasoning during the focus group sessions that the moderator might have overlooked. Both the moderator and co-moderators took notes during the sessions. The interviews were conducted using an interview guide (Table 1). Participants were encouraged to freely discuss their thoughts, views, and experiences on the topics.

**Table 1.** Focus Group Questions.

Main questions
Tell us about the men who come here seeking sexual health care
What is it like to meet men seeking sexual health care?
What is masculinity to you?
What is your perception of the men that come here?
Are there qualities that you consider to be masculine or un-masculine?

To extract more details, further explanations, and examples, the moderator used probes and asked follow-up questions (e.g., *do you all agree with this, can you give examples, or what do the rest of you think about this*). The interview guide was pilot-tested in focus groups at two clinics (four and five participants at a youth clinic and a sexual health clinic, respectively). The questions were not changed after the pilot tests, but probes and follow-up questions were adjusted to ensure inclusion and further discussion. Data from the pilot interviews were not included in the analysis or the results. All interviews were audio-recorded and transcribed verbatim by a professional transcribing firm. All transcripts were scrutinized and corrected for any errors by the first author to ensure high quality and accuracy.

### Data Analysis

After completing the data collection, the data were analyzed for manifest and latent content, using content analysis as described by Graneheim and Lundman (2004) and Graneheim et al. (2017). The analysis was conducted by two authors (T.P. and E.T.). E.T. had no prior involvement in the study and could therefore approach the data openly and from a new perspective. Initially, each focus group was treated as a separate unit of analysis and the data read through several times to get a sense of the text and the content. The transcripts were then imported into NVivo 11.3.1, which is qualitative data analysis software. In NVivo, the data were scrutinized to determine how they related to the aim of the study and the research questions. Relevant data were extracted as meaning units and then condensed and coded. Codes were then aggregated into higher orders of abstraction, that is, subthemes. These in turn were then organized into themes that unified the content of the subthemes (Table 2). This was an iterative process involving going back to previous phases and lower orders of abstraction until the subthemes and themes became clearer. Every step involved discussion and consensus between the analysts.

**Table 2.** Example of Analysis of Descriptive Theme.

Meaning unit	Condensation	Code	Subtheme	Theme
I think that we have a, actually, somewhere a very strong image of what is masculine. But we have a hard time describing it	Masculinity is clearly understood on one level, but cannot be described	Understood but indescribable	Clear notions of masculinity need not translate to an ability to describe it	Contradictory masculinity—elusive but clear

After formulating three descriptive themes, an underlying or theme of meaning emerged. The process of analyzing the underlying theme started with identifying common patterns in the descriptive themes, their subthemes, and meaning units (Graneheim et al., 2017), with an emphasis on how notions of masculinity were expressed. Finally, the raw data were revisited to challenge and confirm the themes until consensus was reached within the author group about the naming and content of each theme.

Notes from the interviews were revisited throughout the analysis process. Preliminary findings were regularly reviewed, discussed, and revised between the authors.

## Results

### Participants

Seven focus groups were carried out with four to six participants per group. A summary of participant demographics and catchment distribution is found in Table 3. From qualitative content analysis of the data, three descriptive themes and one theme of meaning were identified. Table 4 provides a summary of themes, examples of subthemes, and illustrative quotes.

**Table 3.** Study Population and Catchment Distribution.

Characteristics	
No. of participants	35
Gender, *n* (%)	Female 24 (68.6%), Male 11 (31.4%)
Age range	29–71 years
Professions	Assistant physician, Counselor/Social worker, General practitioner, Midwife, Nurse, Assistant nurse, and Psychologist
Types of clinic (*n*)	Primary health care clinic (1), Venereology clinic (1), Youth clinic (3), Reproductive clinic (1), Men’s sexual health clinic (1)
Catchment areas (*n*)	Inner-city (2), Suburbs (2), Smaller towns (2), Rural area (1)

**Table 4.** Themes, Examples of Subthemes, and Illustrative Quotes.

Themes	Examples of subthemes	Illustrative quotes
Descriptive Theme 1: Contradictory Masculinity—Elusive but Clear	Elusive: Masculinity is a fluid construct Masculinity is unknown and eludes explanation Patients do not share a common masculinity Clear: It is clear what masculine is not Men are associated with specific attributes Normative masculinity is easy to describe	“Yes, because I don’t even know what it [masculinity] is because it is changeable . . . I don’t see it as something that has its own core.” “Wow, how difficult it was to define what masculinity is. No, that was too complex to answer.” “I have to say, it is a bit hard to grasp those [men] I have seen [as patients] as I group.” “[If] You are indecisive and not accountable for your word. I would find that a bit unmanly.” “Generally I would say that guys are more boisterous and loud.” “It is much easier to talk about masculinity and stereotypes or negative [aspects], a bit.”
Descriptive Theme 2: Sexual Health Care is a Social Place where Men and Masculinities can be Challenging	Experiencing unwanted sexual tension Feeling insecure and inadequately prepared (for interactions with men) Seeking sexual health is doing un-masculinity	“It can be hard to avoid a heterosexual attraction in the room if the man [patient] is close [to me] in age” *A*: “But I feel that I am very much lacking in how I should treat them [men] in a good way, because I give women much better care than guys. Definitely.” *B*: “I agree with that, I feel exactly the same.” “The ideal image [of masculinity] that we have, that I addressed earlier, it shatters quickly when we meet those [men] who come here.”
Descriptive Theme 3: Regarding Masculinity as Irrelevant—a Difficult Ambition to Achieve	Gender isn’t relevant for me as a professional Professionals have unaware notions that show through (in their profession), even though they should not Unwilling/unable to categorize men as a group	“[Y]ou have to go on what kind of person they are, not what their gender is.” “If we all spontaneously say that yes, we treat men differently, or genders differently, then surly they sense that, somehow, even though we don’t want them too.” “It is difficult to only speak of men. Yes. Or are they somehow different [from other patients], it doesn’t feel that way, I don’t think so.”
Theme of Meaning: Notions of Masculinity are Situated Relationally	Heterosexual romantic relationships at the core of masculinity Masculinity defined as a contact advertisement My male partner/family member exemplifies masculinity	“[I]f there is a man and a women and they are attracted [to each other], if they are oriented that way, then that becomes so darn good, then it is love, but that is because there are opposites.” “I often say, I am straight, well almost, and when I am looking for a man in that way I usually say that I want a man that is secure enough in his masculinity to dare to be feminine.” “[I have] a father, and older brother and I had a fantastic good man and have a new good man who is, there is something very good, there is much of this warmth, that warm goodness you put your cheek against . . . a bit of sweat, stubble and joy, lightness, happiness, that is masculinity.”

### Theme 1: Contradictory Masculinity—Elusive but Clear

The first descriptive theme showed a contradiction in how notions of masculinity were expressed. Masculinity seemed to be elusive in the sense that it was difficult or even impossible to describe as a phenomenon or concept. On the contrary, participants gave clear examples and explanations of traits, behaviors, and qualities deemed as masculine or un-masculine.

### Elusive Masculinity

. . . Oh God, wow, here I am at 51 years old and I don’t have a clue about what masculinity is, that is weird.

When asked to describe masculinity, we observed silence as a response, explained by some as a question that was too difficult to answer. Different reasons were given for this inability or hesitancy. Masculinity was said to be too hard to describe or explain. Masculinity was described as something unknown, either through statements about not knowing what it was or through describing it as indefinable. While some said outright that defining or explaining masculinity was impossible to do, others came to this conclusion while trying to describe their notions. The more they tried to think about it, the harder it became to grasp, and for some it was difficult to describe at all. These difficulties in understanding and describing masculinity were surprising or even a little shocking to the participants.

A reason for the inability to describe masculinity was its perceived vast scope: It was seen as too broad or complex to describe and there were no limits to what it could include. Participants argued that masculinity used to be more distinct and easier to describe and that it had changed over the past few decades to something that is difficult to define. In this way, masculinity was thought of as being flexible or imprecise, as a varied or fluid construct. This was exemplified by describing masculinity as changeable over time, settings, and groups and it was therefore seen as impossible to give any exact descriptions of it:

Back in the days in the 70s then of course there were more [clear] images of what was masculine and feminine. [If you are] Feminine then you wear high heels, and it is physical attributes, and you would wear makeup and you please [others]. And masculinity would be more taking initiative. But to me that was the past.

One approach to the difficulties in describing and understanding masculinity was by talking about male patients as a group. Participants stated that their patients do not share a common form of masculinity and that it was difficult to separate men from other patients (i.e., women and transpersons). Masculinity was also perceived as having a private nature, as it pertained to one’s private life or personal relations, which made descriptions and explanations of masculinity difficult. Viewing masculinity as private set up an obstacle for describing it in a professional context:

Yes, I also think it is a bit difficult [to say what masculinity is]. Perhaps it is easy for it to get a bit private like that.

When reflecting on the difficulties in describing masculinity, participants were puzzled that it had not previously been discussed at their workplace, despite perceptions of having an open climate of dialog at work.

Masculinity was not thought of as separate from other identities or social positions. It was described as intersecting with societal norms relating to sexuality, sex/gender, culture, ethnicity, and age and was therefore perceived to be complex:

And, it [masculinity] is changeable and filtered through class-filters, cultural filters or ethnic [filters] . . . Because it, masculinity, is filtered through those norms that exists in society.

The seeming elusiveness of the concept or the phenomenon as a whole was not necessarily an indication of a lack of clear ideas of what masculinity was. These difficulties could be due to problems with translating notions of masculinity into words:

It is very clear, I think, that we, and I, that is me absolutely as well, have a very clear image of what is masculine. But damn, it is hard to express it.

### Clear Masculinity

In contrast to the elusiveness of masculinity as a concept or phenomenon, participants gave clear examples of traits, qualities, and behaviors that represented masculinity. These included (a) having a direct style of communication; (b) being secure, strong, and not showing weakness; (c) not letting emotions interfere with one’s professionalism; and (d) being robust, hardy, and active. In some ways, it seemed uncomplicated to determine whether specific traits, qualities, or behaviors were associated with masculinity. Participants were aware that their examples were partly based on stereotypical, normative, or traditional views on masculinity and this was seen as problematic.

Masculinity was either primarily associated with positive attributes, traits, and behaviors or with negative stereotypes. Positive examples were sometimes expressed in generalized terms, but tended to be associated with specific men. Examples included being respectful, gender-equal, unafraid to be vulnerable, and able to communicate about feelings. In cases where masculinity had primarily negative associations, positive examples were only thought of when they were asked whether masculinity was purely negative:

Person A: “Are there no positives then, that are associated with masculinity?”

Person B: “I don’t know, are there?”

Person A: “Security, affect-stability or something similar.”

Person C: “Drive.”

Person A: “Drive, yes, that’s right. That’s coded masculine, I think.”

Person D: “Active, passive, the men are active, I think.”

Person A: “Yes.”

Person C: “Responsible, providing.”

Masculinity was also described by examples of and in comparison with un-masculinity. Participants expressed clear opinions about what they thought of as un-masculine, such as being camp, effeminate, being feminine, wanting to be pampered, being too involved in a pregnancy, being fake, being gender queer, indecisiveness, being a bad father, not keeping your word, or being giggly:

[W]hen men come [to the clinic] that are very. . . the hair is dyed and there is a lot of perfume and there are piercings and everything and . . . then I feel, oh my god, this isn’t masculine.

Participants also reflected that they found it easier to exemplify and describe un-masculinity than masculinity and that masculinity was better understood in comparison with un-masculinity. Participants described men in comparison with women, but masculinity was primarily understood in contrast with un-masculinity rather than femininity.

### Theme 2: Sexual Health Care Is a Social Place Where Men and Masculinities Can Be Challenging

The second descriptive theme showed that the participants perceived men and masculinity as potentially problematic or challenging within the sexual health care setting as a social place. Participants felt ill-equipped to treat men’s sexual health due to lack of experience, training, or education and believed that their professional demeanor and care suffered in interactions with men. Challenges could arise from both HCPs’ and patients’ perspectives. In either case, HCPs had to deal with and try to handle these challenges. Masculinity was seen as a potential barrier to sexual health. Participants who lacked training and organizational prerequisites said that it affected their views on working with men’s sexual health, including their perception of men as patients:

[T]his is a place where masculinity becomes something difficult.

The notion of men and masculinities as a potential barrier to adequate sexual health care was founded on the relationship between the patient and the HCP. These notions were based on experiences of meeting men as patients and on realizations about the importance of the HCPs’ own identities during these interactions. Working with men’s sexual health was associated with having to manage sexually charged situations and unwanted sexual tensions. This was particularly clear for female HCPs as heterosexual social norms were perceived as affecting professional–patient interactions. Interactions, such as physical examinations, could blur the line between professional and private. Genital examinations were described as private and intimate situations for both patients and HCPs. The participants’ identity and appearance influenced the interactions, given that factors such as gender, age, and attractiveness could either increase or reduce sexual tension. Being the “same age” as a patient was thought to make communication about sexuality awkward and made men embarrassed, whereas being older was associated with being less sexually threatening and with the ability to create a safer and more secure situation for the patient. Feeling and conveying security, and being experienced in one’s professional field were described as alleviating unwanted sexual tension:

Yes, I agree since I am one of the elders [at the clinic] I don’t have any difficulties with any age. And I also feel, which I didn’t at all then of course, when I was younger it was completely different, but I feel that younger [men] can. They don’t have to relate to me as a sexual object as much. And, and yes, they do, they can but I don’t care and I can handle it.

Organizational prerequisites, professional training, and experience with men’s sexual health varied greatly between participants and clinics. Although all participants worked at clinics providing sexual health care for men, participants at some clinics said that they lacked sufficient organizational prerequisites and support from management regarding this aim. Requests for further education in the field were denied. Those with existing expertise felt it was undervalued and some medical records did not accept men’s social security numbers (the sex of an individual can be ascertained from Swedish social security numbers). The situation for men in need of sexual health care was described as a “black hole”: Men lack access to services and the field has a shortage of expertise. The situation for HCPs working with men’s sexual health was described as “living in a twilight zone” and working with men’s sexual health was described as doing something “forbidden.” Counseling on sexuality with men was done “behind the scenes” and then reported as STI prevention in medical records. However, it was still seen as a good thing when men came to the clinic as they believed that men seek sexual health services infrequently. Strategies to deal with insecurities or a lack of training included referring men to colleagues with more training, to other clinics, or trying their best to help.

Certain expressions of masculinity were described as problematic in a sexual health care setting. These were behaviors found to be provoking, disturbing, or threatening, such as domineering, rude, and belittling behavior. Dominance and belittling were perceived as behaviors used by men to avoid sexual health issues. Examples of this were men treating sexual health issues as a joke, changing the subject, asserting that the sexual health issue “is the way it is,” raising their voice, or banging the table. Other things that provoked participants were men displaying what was considered un-ethical sexual conduct (e.g., cheating on a pregnant wife), men not having a sense of humor, men addressing female HCPs as “sweetie,” and men appearing as un-masculine (e.g., men with make-up). Being provoked or becoming angry could challenge the professional role, and hence there was a need to put on metaphorical “armor”:

Person A: “I don’t get as provoked at work, but can be violently provoked privately.”

Person B: “I don’t get provoked at work, either.”

Person A: “That is, I don’t get provoked by men that. . . No, I don’t think so, I get. . .”

Person B: “You put on your armor.”

Although the “armor” got stronger with experience, participants agreed that reactions still seeped through. Meeting men could be positive and rewarding, but some expressions and behaviors that were perceived as masculine were hard to deal with professionally, such as violence, aggression, and hate. Male aggression was perceived as more threatening than aggression coming from transpersons or women. Certain identities and groups of men were portrayed as more difficult to interact with and relate to. Men with non-Swedish culture or religious background were described as problematic regarding how they perceived and related to gender and sexuality, as these men were regarded as not sharing a “Swedish” perception on gender equality. Rural, older, and upper-class men were more challenging and harder to relate to as they were perceived as more difficult to relate to and communicate with. The image of an ideal patient was a Swedish, young, urban man from working- or middle-class background, having a “correct” view on gender equality, and a good ability to communicate.

Altering problematic masculinity was seen by some as a professional mission when working with men’s sexual health. Participants expressed opinions that masculinity should be changed, counteracted, or abolished. From this point of view, masculinity was thought of as something that legitimized violence, control, and dominance, and as hindering men from being vulnerable and help-seeking. Men seeking sexual health care would also get help with shaping, teaching, rearing, and creating the right kind of masculinity, which ideally meant not having problems with help-seeking and communicating about sexuality. In one interview, the clinic was described as a “bubble” where masculinity could be destroyed. Men that raised concerns about sexual health or that came to a clinic were described as being brave enough to show weakness or vulnerability. The act of seeking sexual health services was described as doing something outside of masculinity norms, something that could make the setting difficult for men. Having sexual health issues, being care-seeking, and communicating about sexual health issues were all perceived as ways of doing un-masculinity. Participants expressed pride in the fact that men did something potentially un-masculine by coming to their clinic to seek help with their sexual health issues:

I probably think about the men, men that come here seeking help with their sexual health. They are doing un-masculinity. They are performing something un-masculine . . . you have an erectile dysfunction, and you are talking about it and you are asking for help. That is three things at once.

### Theme 3: Regarding Masculinity as Irrelevant—a Difficult Ambition to Achieve

The third descriptive theme deals with the participants’ ambitions to disregard gender and masculinity as a professional approach to patients. This ambition could be difficult to achieve. A professional approach to men and masculinity was described as disregarding patients’ sex and gender, as these were seen as irrelevant. Instead, patients were described as being human, patients, or individuals rather than being men or masculine. Although gendered notions were acknowledged on a group level, these notions were not deemed to be applicable or relevant when meeting individual patients:

So, regardless of whether it’s a man or a woman I think [of the person as a] patient. Patient. Yes. That is what I do [professionally], it is patients, it is not men or women, it is patients. Really. And then you don’t think about that. In my opinion. . .

Gender-neutrality was described as being open-minded with regard to patients; it was thought of as not having preconceived notions about them and as having a neutral professional demeanor, that is, not regarding patients’ gender when giving information or answering questions. Striving for neutrality and disregarding patients’ sex and gender were ongoing efforts that demanded constant awareness. Gender-neutrality was either seen as a learning process, a result of clinical experience, or as a prerequisite for working clinically with sexual health:

[I] always have to think about how I answer, so I don’t make assumptions [and answer] guys in a specific way or a girl in a different way. Truly being neutral and meeting that specific patient as an individual and trying to disregard how I would answer [because the patient is] a guy.

Although gender-neutrality was seen as a professional approach, it was not always possible to achieve. Participants said that patients’ sex and gender could not and should not be completely disregarded, and that they probably failed to be gender-neutral because they were unaware of their own gender-biases. These were thought to affect their professional demeanor and their treatment of men. Participants thought it likely that men sense that HCPs treat and interact with them differently than other patients.

The participants showed awareness of transgendered and nonbinary patients and an understanding that gender expressions are not always a clear indication of a person’s gender identity. This was yet another reason to be gender-neutral in interactions with patients. Participants described becoming aware of their need to sort patients into gendered categories when meeting patients whose gender was not immediately identifiable by gender expression. Categorizing patients went against their ambition of being gender-neutral.

The participants described a difference between their private and professional notions of masculinity. Sexual health was thought of as an area where distinctions between being private and being professional were more important than in other areas of health care, although this distinction was not always achievable. Private and professional aspects would seep into each other, but part of being a professional was being able to disregard one’s private notions. Keeping private notions separate was thought to be easier in relation to certain groups of patients, such as young men, who were not regarded as men but as youths. Keeping the notions apart was a challenge to participants, who felt that their education, training, and previous work experiences had not given them a basis for developing a professional approach to men and masculinity. Consequently, they had to rely on private notions of masculinity when meeting men as patients.

### Theme of Meaning: Notions of Masculinity Are Situated Relationally

The theme of meaning was a relational and sometimes sexualized understanding of masculinity. Personal relationships were used as a frame for participants’ notions. In this sense, masculinity was partly constructed in relation to oneself and to others, that is, situated in the context of intimate and private relationships. The theme of meaning runs through the three descriptive themes. Describing masculinity was difficult partly because doing so was too private or personal, and personal relationships were used to illustrate masculine traits, behaviors, and qualities (Theme 1). Participants said that their identities and expressions, such as gender, age, and attractiveness, were important factors in interactions with men seeking sexual health care, and sexual tensions were ascribed to these interactions (Theme 2). A relational perspective on masculinity seemed to underlie the reasoning of why gender-neutrality was viewed as professionality and why participants distanced themselves from describing patients as men or masculine as this could blur the lines between professional and private (Theme 3):

What masculinity is, yes, that is a very hard question. Then I think about my boyfriend. He is very good at cooking. He is tender, humble, funny. He has a very attractive penis.

Relationships to partners, spouses, fathers, and children were at the center of the descriptions of men and masculinities. Positive qualities of participants close relations were used to illustrate views on masculinity. Being a good father to one’s children was used as an example of something inherently masculine. When masculinity was portrayed negatively, it was made clear that descriptions or examples were not based on the participant’s personal relationship:

[Masculinity] is also an entitled authority, if I should say something about negative sides, I am not speaking about the men that are close to me, but. . .

Definitions and explanations of masculinity were based on personal preferences in romantic and sexual relationships. The concepts of security, safety, and stability were central in these preferences, such as a man’s ability to provide security for his wife. The good qualities in actual or potential relations were used as synonyms for masculinity. Traits and behaviors that were described as un-masculine were depicted as clearly unattractive. In this sense, masculinity was situated within experienced or hypothetical heterosexual relationships:

Masculinity is, is something I am attracted [to], warmth, caring and safety.

That masculinity that was situated in heterosexual relationships made it complicated to combine notions of masculinity with homosexuality:

It gets a little difficult [to talk about masculinity] when we talk about homosexuality and such, because then it becomes a bit slippery [to define masculinity].

It seemed difficult to describe masculinity without sexualizing men, thus displaying a sexual component in the understanding of the concepts. The associated values were to some extent sexualized and sexuality was assumed to be an integral part of masculinity. Men’s sexuality was described as mechanical or technical and as something that should “just work.” If it did not, due to some health issue, it was described as being a big problem for men.

## Discussion

To explore notions about men and masculinity among HCPs working with men’s sexual health, we conducted seven focus group interviews with 35 participants. There were a number of contradictions in how notions about men and masculinities were expressed. Participants said that it was difficult or even impossible to describe masculinity as a phenomenon or concept, but gave clear examples of specific masculine and un-masculine traits, behaviors, and qualities. It became clear that participants differentiated between being able to understand masculinity as a phenomenon and giving examples of separate aspects of that phenomenon. The examples largely reflected traditional and stereotypical perceptions of masculinity, suggesting that HCPs’ notions about men as patients were based on normative masculinity. The pattern of how participants expressed these notions appeared as unrelated to the participants’ gender. The participants stated that they probably had biases about men and that men were treated differently from women, transpersons, and nonbinary patients. In the analysis, other potential biases were identified, such as biases against non-Swedish men and men with a rural background. Previous research reports that a negative perception of a group of patients can relate to how HCPs communicate with patients from a particular group (Street et al., 2007) and that implicit biases among HCPs have a significant relation to patient–provider interactions, treatment decisions, and patient health outcome (Hall et al., 2015). Awareness of one’s notions can be an important first step to counteract implicit biases (Zeidan et al., 2019). If care-seeking men are met by gender-biased and normative notions, this in turn could lessen their inclination to seek help in the future (Govender & Penn-Kekana, 2008; R. Knight et al., 2013) or even affect the quality of care they received (FitzGerald & Hurst, 2017).

Sweden, like other Nordic countries, has been described as progressive in the area of gender equality (Larsen et al., 2021), ranking first on the EU’s Gender Equality Index in 2020 (European Institute for Gender Equality [EIGE], 2020). Previous research suggests that normative masculinity in Sweden is changing to include ideas of gender equality and being a caring and present father (Johansson & Klinth, 2008). Just as masculinity may vary between contexts and over time (Brod, 1987), we saw patterns of variation in whether participants mainly focused on negative or positive associations with masculinity, such as associating gender equality with masculinity and perceiving that as desirable. Such variations could be important for how men as patients are perceived by health care providers. If, as this study suggests, HCP gender notions and norms are reflected in their professional demeanor and treatment of patients, then awareness of this and changing notions of normative masculinity could be vital steps in improving and professionalizing interactions with men seeking sexual health care.

According to the theory of Schippers (2007), masculinity is a combination of practices, characteristics, and embodiment that have far-reaching social effects, as well as a “social location” that a person can inhabit through practices. Social location in this sense is not strictly a place, but a social position that can be embodied and practiced in social interactions. We found that masculinity in the sexual health care setting is a social place, in the same sense that Schippers defines social location, and that this social place is potentially problematic from both HCPs’ and patients’ perspectives. This social place was associated with aggression, dominance, belittling behaviors, and unwanted sexual tension. Male patients were also assumed to be heterosexual. These assumptions reflect societal expectations on men as straight in a feminized arena, the sexual health care setting, in a similar way that male nurses experience sexualized assumptions from female students, staff, patients, and families (Le Blanc, 2016). The participants in the present study considered sexual health care to be a challenging social location for men as patients, as the act of seeking sexual health care, having issues with one’s sexuality, and communicating about such problems were all thought of as ways of doing un-masculinity. Thus, patients’ masculinity was seen as a barrier to seeking health care. This challenges the notion that all health care is andronormative (Holge-Hazelton & Malterud, 2009), that is, designed for a male patient. The concept of andronormativity implies that normality and neutrality in health care and medicine are constructed in ways that make men the normal and expected patients. Based on the results of this study, sexual health care seems to be an exception. HCP strategies for dealing with masculinity as a problematic and challenging social location were to metaphorically put on armor and to work to change, destroy, or cultivate the right kind of masculinity in patients. By perceiving masculinity as a social location in line with Schippers (2007) and exploring it within a particular setting, in relation to broader structures, to other identities and practices, particularly which masculine practices are viewed as disruptive or un-masculine, we can begin to understand how gender operates within the setting.

Patients’ masculinity was said to collapse during interactions with HCPs and care-seeking men’s masculinity did not live up to participants’ ideals regarding masculinity. However, men who seek care for sexual health were also described as bold and brave, which seems to be contradictory. On one hand, HCPs uphold normative masculinity, and on the other hand, they criticize it. Seeking care for sexual health is described as performing both un-masculinity and doing something traditionally masculine, that is, being brave and bold. In this sense, participants seemed to both endorse and criticize normative masculinity. This reflects previous research (Seymour-Smith et al., 2002) that reports that HCP discourses both criticize normative masculinity for its negative health impacts on men and protect it.

Participants said that lack of training, experience, and organizational prerequisites affected their views on working with men’s sexual health. It is difficult to say how this may affect their perceptions of men and masculinity. We have shown that masculinity is a potentially problematic social location in sexual health care, but whether this should be partly understood as a consequence of participants lacking sufficient training and organizational support cannot be concluded from this qualitative study. Gender-sensitivity training can decrease gender bias and improve attitudes and practices in health care (Lindsay et al., 2019). However, future follow-up studies regarding gender awareness need to be conducted for HCPs with different educational backgrounds.

Previous research has suggested that disregarding gender can be “a particularly powerful way of doing gender” (Holge-Hazelton & Malterud, 2009) and that assuming sameness between genders may be a form of gender bias (Risberg et al., 2009) that may contribute to upholding existing gender inequalities in health care. This study identified that participants strive to disregard masculinity by being gender-neutral in their approach to patients. By striving for gender-neutrality rather than gender awareness, HCPs risk perpetuating and reproducing normative masculinity that could create barriers to adequate care for men seeking sexual health care and for HCPs when trying to provide such care.

Notions and experiences of masculinity were partly constructed in relation to oneself and to others. Descriptions of masculinity were situated in real or hypothetical personal and intimate relationships. Personal preferences in romantic or sexual relationships were used to define and exemplify masculinity. Participants ascribed heterosexual tension to interactions between female HCPs and men as patients. HCP identities and expressions could alleviate or aggravate this. Professional and gender-sensitive interpersonal dynamics between HCPs and patients have been identified as important for the improvement of men’s health and the likelihood of care-seeking, particularly regarding potentially sensitive issues such as sexual health (Leone et al., 2021). Our findings suggest that HCPs use private and personal notions of men and masculinity in relation to men seeking sexual health care, that is, they have not incorporated gender awareness in how they describe men and masculinity. This could be due to a lack of professional training.

### Limitations

Although these findings contribute to the understanding of HCP notions of gender and masculinity and the importance of gender awareness in medicine in general and within sexual health care specifically, they must be perceived in the light of the study’s limitations. First, we assumed that participants would be more comfortable talking about the research questions in a familiar setting and with co-workers. That their notions of masculinity turned out to be partly situated relationally and even sexualized could have hindered participants from speaking freely and truthfully in the focus group format. Second, focus groups can foster a group-think mentality and social desirability of wanting to conform with the perceptions and experiences of peers. The interviewer tried to mitigate this by using probes to encourage contradictions and examples. Third, in the recruitment we strived for diversity in both clinical and professional experience as well as catchment areas, but clinics self-selected their participants so data may differ from other clinics. Fourth, participants were recruited through the SRHR Network that is coordinated by the Knowledge Center for Sexual Health where the first author works. Neither any participants nor the first author hold any formal role in the network and they had no previous knowledge of each other.

### Implications for Education and Interventions

Our findings underscore the importance of further training on men’s sexual health and masculinity. These findings indicate a gap in professional education and training programs. Participants said that they lacked training, provided a low quality care for men, and fell back on private notions of masculinity in interactions with men as patients. Further training on men’s sexual health and masculinities could alleviate barriers to adequate health and help HCPs communicate their perceived challenges regarding masculinity in sexual health care, as these difficulties could be a hindrance to a constructive dialog. This study also illustrates that there are organizational challenges that should be rectified regarding men’s sexual health care, such as certain electronic medical records systems not accepting men’s social security numbers, existing competence in the field being undervalued, and requests for further education being denied. To address these challenges, interventions will need to occur on a structural level. To enable this, future studies could explore health care policymakers’ and decision-makers’ perceptions of men’s sexual health. This study was conducted in a Scandinavian context and HCPs may have been influenced by societal view on gender equality and sexual health. In spite of this, we found that HCP seem to need further training. It can be expected that this need is present also in other cultures and countries. Thus, this work can still inform many groups, including those in other societies with other perceptions on gender and sexuality.

To verify the transferability of the results, studies in other cultural contexts are needed.

## Conclusion

This study identified that notions of masculinity among HCPs working with men’s sexual health were simultaneously elusive, but clear enough to exemplify. These examples reflect normative masculinity, that is, culturally dominant idealized and stereotypical notions of masculinity. HCP identities played a large part in their notions of men and masculinity, which highlights the importance of treating masculinity from a structural and professional standpoint so that HCPs do not have to rely on private notions and identities. This study underlines the need for education and training, for HCPs working with men’s sexual health. It also demonstrates the necessity of including gender awareness as part of education for HCPs, to prevent personal notions and norms from being reflected in the professional demeanor and treatment of patients. The study points to deficiencies in organizational prerequisites for providing adequate care for men in need of sexual health care, which suggests there are structural problems and possible biased views on men’s sexual and reproductive health care services and management.
